# Description of a new species of *Ceruchus* MacLeay (Coleoptera, Lucanidae), with taxonomic notes on the Chinese species of the genus

**DOI:** 10.3897/zookeys.1289.190703

**Published:** 2026-08-14

**Authors:** Yujie Huang, Yanchen Liu, Yagang Shen, Xueyun Feng, Xia Wan

**Affiliations:** 1 Department of Ecology, School of Resources and Environmental Engineering, Anhui University, 111 Jiulong Road, Hefei, Anhui, China Frontiers Science Center for Molecular Design Breeding, Beijing Key Laboratory of Crop Genetic Improvement, College of Agronomy and Biotechnology, China Agricultural University Beijing China; 2 Frontiers Science Center for Molecular Design Breeding, Beijing Key Laboratory of Crop Genetic Improvement, College of Agronomy and Biotechnology, China Agricultural University, Beijing 100193, China Department of Ecology, School of Resources and Environmental Engineering, Anhui University Hefei China

**Keywords:** Biogeography, identification key, Palaearctic region, stag beetle, Syndesinae, taxonomy

## Abstract

The generic characters of the stag beetle genus *Ceruchus* MacLeay are comprehensively reviewed and refined based on the examination of type material of most Chinese species. In this study, one new species, *Ceruchus ahualuo* Huang, Liu & Wan, **sp. nov**. is described and illustrated from Yunnan, China. In addition, an updated diagnostic key to all known Chinese species of *Ceruchus* is provided, together with a distribution map of the genus in China.

## Introduction

The genus *Ceruchus* MacLeay, 1819 is currently classified in the tribe Ceruchini of the subfamily Syndesinae (Coleoptera: Lucanidae) (Smith 2006; Bouchard et al. 2011). However, the higher-level taxonomic placement of this genus has long been unstable and controversial. The earliest known species were originally described under the genus *Lucanus* Scopoli, 1763, until MacLeay (1819) established *Ceruchus* based on the type species *Lucanus chrysomelinus* Hochenwarth, 1785. The systematic placement of *Ceruchus* has changed considerably in previous classifications. Benesh (1960) considered the genus to belong to the subfamily Aesalinae MacLeay. However, Holloway (1960, 1968), based mainly on adult external morphology and male genitalia, reclassified *Ceruchus* and placed it together with *Syndesus*, *Sinodendron*, and *Psilodon* in Syndesinae. More recently, a molecular phylogenetic analysis further tested the monophyly of Syndesinae and the phylogenetic position of Ceruchini, revealing complex evolutionary relationships that partly challenge traditional morphology-based classifications and suggest that the placement of Ceruchini within Lucanidae remains incompletely resolved (Kim and Farrell 2015).

Earlier taxonomic knowledge of *Ceruchus* was derived mainly from European and North American species, with only a few early records from Asia (Benesh 1960). In recent decades, intensified collecting and taxonomic work in China and adjacent Sino-Himalayan regions has revealed a much richer and previously underdocumented diversity, with several species and new locality records reported from mountainous areas of southwestern and central China (Tanikado and Okuda 1994; Král 1995; Boucher and Král 1997; Okuda 2008; Huang et al. 2011; Huang and Chen 2013, 2017; Huang et al. 2020).

At present, 21 species are recognized in the genus, including 19 extant species and two fossil species, of which 12 have been recorded from China. During a recent examination of Chinese representatives of the genus, we discovered a new species from southwestern China. In this paper, we formally describe and illustrate the new species based on comparative morphology, increasing the number of Chinese species to 13 and the total number of species in the genus to 22. We also provide an updated identification key to the Chinese species of *Ceruchus*, prepared based on our examination of specimens and with reference to Huang and Chen (2017), summarize their distribution in China, and provide mitochondrial COI evidence supporting the separation of the new species from its sympatric close relative.

## Material and methods

The institutions cited in the present paper are indicated by the following abbreviations:

**AHU** Anhui University, Hefei, China.

**MNHN** Museum national d’Histoire naturelle, Paris, France.

**SHNU** Shanghai Normal University, Shanghai, China.

Terminology follows Holloway (1960, 1961). Measurements are given in millimetres (mm). Body length was measured from the apex of the mandibles to the elytral apex.

Morphological observations and measurements were performed using an Olympus SZX7 stereomicroscope. Photographs of the adult specimens and male genitalia were taken with a Canon 5Ds digital camera equipped with a Canon MP-E 65 mm f/2.8 1–5× Macro Photo Lens.

The distribution map of the genus *Ceruchus* was generated in ArcGIS Pro v. 3.7 (Esri, Redlands, California, USA). Geographic information was compiled from published records in Huang et al. (2011), Huang and Chen (2017) and Huang et al. (2020), together with specimen records from our laboratory collections and field survey data. To reduce spatial autocorrelation and visual clustering, the occurrence records were spatially thinned, retaining only one record per species within a 5 km radius. Digital elevation model (DEM) data at a spatial resolution of 30 arc-seconds (c. 1 km) from the WorldClim 2.1 database (Fick and Hijmans 2017) were used as the topographic base map, and provincial administrative boundaries of China were overlaid for geographic reference. The occurrence data underlying this study were deposited in the Global Biodiversity Information Facility (GBIF) and are available at https://doi.org/10.15468/jzbytm (Huang 2026).

For molecular comparison, we newly sequenced two specimens, representing the new species and its sympatrically distributed close relative, *Ceruchus niger* Boucher & Král, 1997. New sequences were submitted to General Biol (Anhui) Co., Ltd (Chuzhou, Anhui, China) for genomic DNA extraction, library preparation, and sequencing. Total genomic DNA was extracted from fresh tissue, and whole-genome paired-end short-read sequencing was performed on an Illumina platform, generating 150 bp paired-end reads and approximately 10 Gb of data per sample. Mitochondrial candidate scaffolds were recovered from the whole-genome reads using GetOrganelle v. 1.7.7.1 under the animal mitochondrial mode. The standard DNA barcode fragment of mitochondrial cytochrome *c* oxidase subunit I (COI) was identified from the mitochondrial candidate scaffolds by BLAST searches against reference sequences and subsequently extracted for genetic distance analysis and tree reconstruction. Additional COI sequences of *Ceruchus* species were downloaded from GenBank. The COI sequence of *Sinodendron yunnanense* was extracted from its mitochondrial genome sequence downloaded from GenBank and used as the outgroup. All sequences were aligned using MAFFT v. 7.526 with automatic direction adjustment. Pairwise genetic distances among the sampled *Ceruchus* species were calculated under the Kimura 2-parameter model. A maximum-likelihood tree was reconstructed in IQ-TREE v. 3.1.3 using the best-fit substitution model selected by ModelFinder. Branch support was assessed using 1000 ultrafast bootstrap replicates.

Information on all sequences used in this study is summarized in Table 1.

**Table 1.** List of specimens used in the analysis, including sampling localities, GenBank accession code, and the origin of data.

**Species**	**Sample Locality**	**GenBank Accession**	**Source**
*Ceruchus ahualuo* sp. nov.	Yunnan, China	PZ603445.1	This study
*Ceruchus niger*	Yunnan, China	PZ603446.1	This study
*Ceruchus lignarius*	Gunma, Japan	AB427048.1	GenBank
*Ceruchus minor*	Sichuan, China	LC511010.1	GenBank
*Ceruchus minor*	Sichuan, China	LC511011.1	GenBank
*Sinodendron yunnanense*	Yunnan, China	KP735804.1	GenBank

## Results

### Taxonomic account

**Tribe Ceruchini**

#### Genus Ceruchus

Taxon classificationAnimaliaColeopteraLucanidae

MacLeay, 1819

91B5AD32-B5B4-5A03-B419-43EEED2B3DDA

Ceruchus MacLeay, 1819: 115. Type species: Lucanus chrysomelinus Hochenwarth, 1785.
Platycerus Latreille, 1807: 133. Preoccupied name: Platycerus Geoffroy, 1762 in Lucanidae.
Tarandus Megerle: Dejean, 1837: 194. Nomen nudum in Dejean’s Catalogue des coléoptères de la collection de M. le comte Dejean.

##### Material examined.

*Ceruchus sinensis* Nagel, 1933: ***Holotype***. China • 1 ♂; Yunnan Province, near Mt Frances Gardner, west of Mekong, elev. 2438 m, 9.VII.1922; Prof. J.W. Gregory leg.; MNHN. ***Non-type material***. China • 1 ♂; Yunnan Province, Weixi County, Baijixun Township, 1.IV.2021; Zhiwen Yu leg.; AHU.

*Ceruchus minor* Tanikado & Okuda, 1994: ***Paratype***. China • 1 ♀; Shaanxi Province, Qinling Mountains, Huxian, elev. 1400–1600 m, 33.9167°N, 108.5000°E, 27.VI.1993; Tabana leg.; MNHN. ***Non-type material***. China • 1 ♂; Shaanxi Province, Qinling Mountains, Changan, elev. 1400–1600 m, 11–24.IV.1993; M. Tabana leg.; MNHN. China • 3 ♂ 2 ♀; Shaanxi Province, Mei County, Honghegu, elev. 1600 m, III.2025; Yanchen Liu leg.; AHU.

*Ceruchus deuvei* Boucher & Král, 1997: ***Paratype***. China • 1 ♀; Sichuan Province, Mts. Siguniang, Huangqiao-gou Valley, southwest of Lixian, elev. c. 2500 m, deciduous forest; MNHN. ***Non-type material***. China • 1 ♂; Sichuan Province, Niuzhihe Fl., Pusiun, elev. 2200 m, 16.VI.2000; Belousov leg.; MNHN.

*Ceruchus niger* Boucher & Král, 1997: ***Holotype***. China • 1 ♂; Yunnan Province, Zhongdian, elev. 3300 m, Abies forest, VII; Th. Deuve leg.; MNHN. ***Non-type material***. China • 4 ♂ 3 ♀; Yunnan Province, Weixi County, Zhonglu Township, III.2021; Yanchen Liu leg.; AHU. China • 2 ♂ 1 ♀; Yunnan, Shangri-La City, VI.2018; collector unknown; AHU. China • 2 ♂; Yunnan Province, Weixi County, Zhonglu Township, III.2026; Yujie Huang leg.; AHU.

*Ceruchus yangi* Huang, Imura & Chen, 2011: ***Holotype***. China • 1 ♂; Guizhou Province, Jiangkou County, Mt Fanjingshan, elev. c. 2000 m, 25.X.2009; X.-D. Yang leg.; SHNU. ***Non-type material***. China • 2 ♂; Chongqing, Nanchuan District, Mt Jinfoshan, elev. 1855 m, III.2025; collector unknown; AHU. China • 2 ♂ 1 ♀; Guizhou Province, Leishan County, Mt Leigongshan, elev. 2000 m, IV.2021; Yanchen Liu leg.; AHU.

*Ceruchus yingqii* Huang & Chen, 2013: ***Holotype***. China • 1 ♂; Sichuan Province, Xiaojin County, Rilong Township, Changping-gou Valley, elev. 3600 m, 6.VIII.2011; H. Huang leg.; SHNU.

*Ceruchus ganyanae* Huang & Chen, 2017: ***Holotype***. China • 1 ♂; Sichuan Province, Muli County, near Kangwu Pass, elev. c. 3200 m, VI.2011; Ganyan Yang leg.; SHNU.

*Ceruchus motuoensis* Huang, Chen, Tao & Xiao, 2020: ***Holotype***. China • 1 ♂; Xizang, Motuo County, 62k, elev. 2754 m, 13.XI.2019; R.-C. Tao & C.-M. Xiao leg.; SHNU.

*Ceruchus tabanai* Okuda, 2008: ***Non-type material***. China • 1 ♂ 1 ♀; Sichuan Province, Puge County Mt Luojishan, III.2026; Yanchen Liu leg.; AHU.

*Ceruchus reginae* Boucher & Král, 1997: ***Non-type material***. China • 1 ♂; Yunnan Province, Fugong County, Shiyueliang Township, elev. 2900 m, 15.III.2021; Yanchen Liu leg.; AHU.

##### Description.

Generic characters. Members of the genus *Ceruchus* are relatively small. Sexual dimorphism distinct. Body mostly reddish-brown or dark brown. Dorsum slightly convex, relatively smooth, and sparsely setose. Venter possesses more pronounced short setae. Compound eyes small and entire, lacking canthus. Antennae stout and not distinctly geniculate; scape relatively stout and curved, forming an angle greater than 90 degrees with pedicel. Pedicel longer than antennomeres 3–7; antennomeres 8–10 dilated, forming a 3–segmented club. Both maxillary and labial palps considerably long; maxillary palps almost equal in length to antennae.

**Male. *Body*** length 9.0–21.5 mm. ***Mandibles*** not longer than combined length of head and pronotum, slightly curved upwards, hollowed on inner-ventral side, and clothed with relatively long, dense setae; apex of mandibles acute; mandibles with a well-developed major tooth near middle or base, curved upward and directed subhorizontally or obliquely forward; one or two small, subquadrate, inwardly curved obtuse denticles present posterior to major tooth toward base; base of ventral mandibular margin usually with a triangular obtuse tooth directed almost vertically downward. ***Head*** relatively broad, with sparse large punctures; an oblique groove formed by a line of punctures present behind each eye; labrum small, usually triangular. ***Pronotum*** subrectangular, with distinct small punctures; anterior margin sinuate, posterior margin nearly straight, and lateral margins nearly parallel. ***Elytra*** with distinct longitudinal striae formed by punctures. ***Legs*** short; tibiae of all legs serrate and armed with sharp denticles; outer apical margin of protibia not bifurcate, bearing only a single acute tooth; inner apical margin with a well-developed spur curved downward; outer apical margins of mesotibia and metatibia without distinct bifurcation, inner apical margins with two asymmetrical outward-curving spurs.

***Male genitalia***. Internal sac not everted from middle to apex; instead, only a slender sac-like structure present within median lobe. Median lobe broad, forming a large plate with a rounded apex. Parameres plate-like and rounded. Basal piece stout, longer than parameres. Median struts variable in thickness and length.

**Female. *Body*** length 9.0–15.5 mm. Mandibles short and small; dorsal margin of mandibular base forming a slightly upward-curved arched plate, apex forming an acute triangular denticle, base obtuse and quadrate, and middle bearing one small denticle, giving arched plate a trifurcate appearance; base of ventral margin without a tooth. Anteromedian portion of vertex with a shallow depression. Pronotum subtrapezoida, distinctly convex and ridge-like, flanked by a small convexity on each side. Setae on inner margin of mandibles, mesofemora, and ventral apex of mesotibiae much less conspicuous and dense than in male. Punctures on head, pronotum, and elytra deeper and denser than in male. Other characters similar to those of male.

***Female genitalia***. Hemisternites subtrapezoidal and plate-like, bearing small styli at apex. Accessory duct long, broad, and apically spherical. Spermatheca often slightly sclerotized; spermathecal duct relatively thin and short, distinctly separated from and narrower than spermatheca; spermathecal gland slender.

##### Distribution.

Holarctic.

#### Ceruchus ahualuo

Taxon classificationAnimaliaColeopteraLucanidae

Huang, Liu & Wan sp. nov.

3EC5E69C-56D6-5658-9976-6C62F28AD62C

https://zoobank.org/AB1D44F9-EA15-4616-99A9-535FB9C8EBAB

Figs 1, 2, 3, 5, 6

##### Chinese common name.

阿花洛角锹甲.

**Figure 1.**
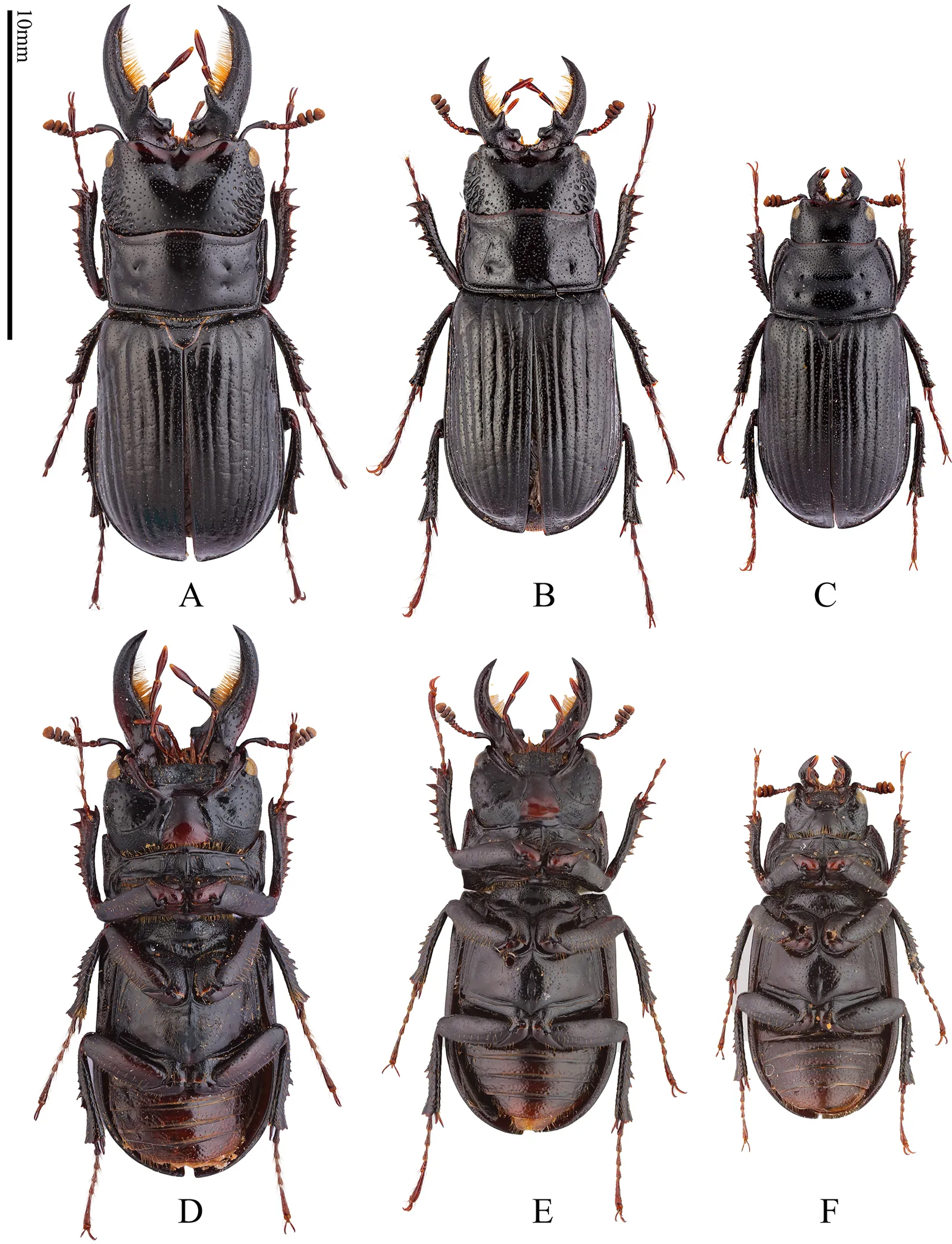
Habitus of *C. ahualuo* sp. nov. **A, D**. Major male (holotype); **B, E**. Minor male (paratype); **C, F**. Female (paratype), dorsal view; **A–C**. Dorsal view; **D, E**. Ventral view. Scale bars: 10 mm.

**Figure 2.**
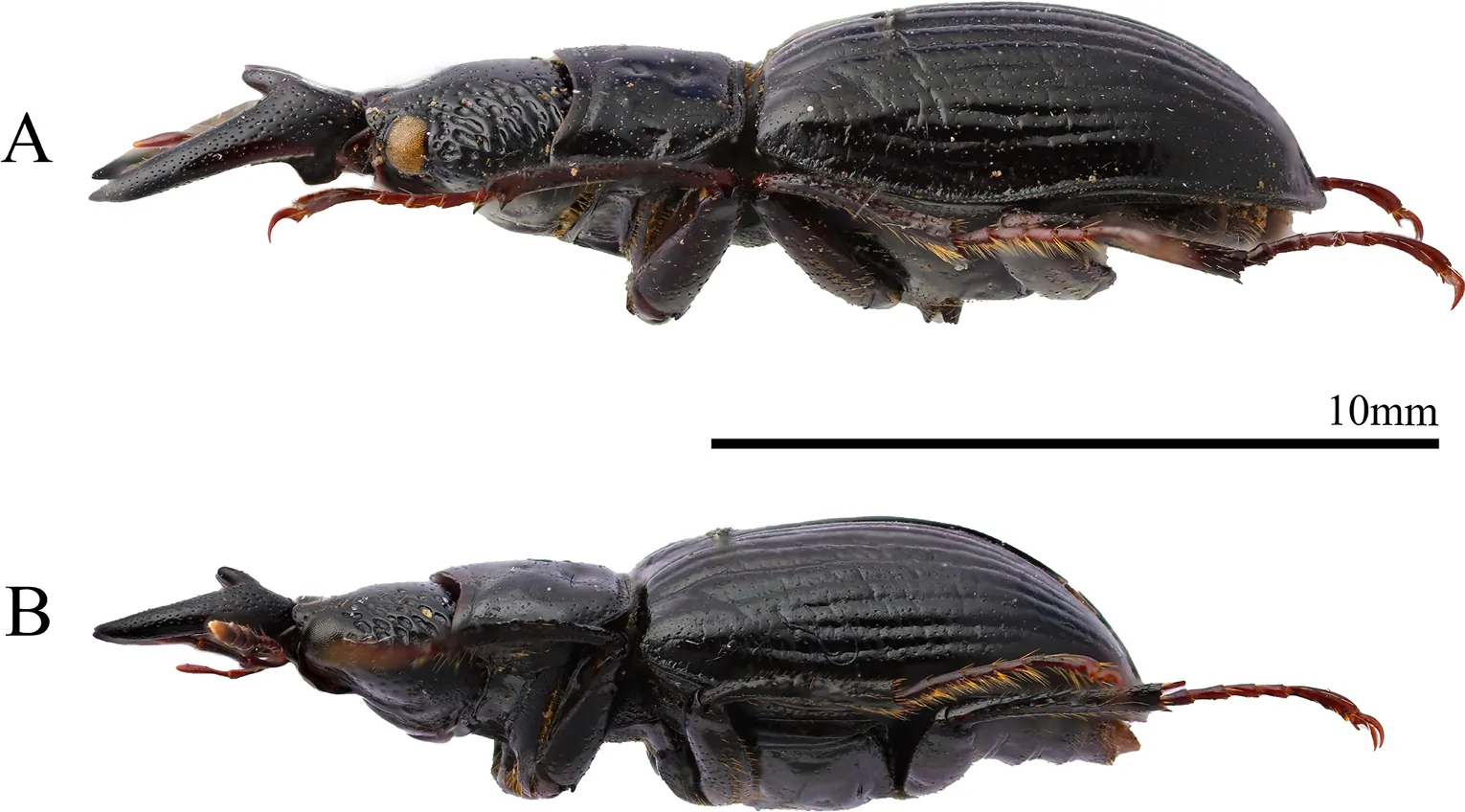
Lateral view of *C. ahualuo* sp. nov. **A**. Major male (holotype); **B**. Minor male (paratype). Scale bars: 10 mm.

**Figure 3.**
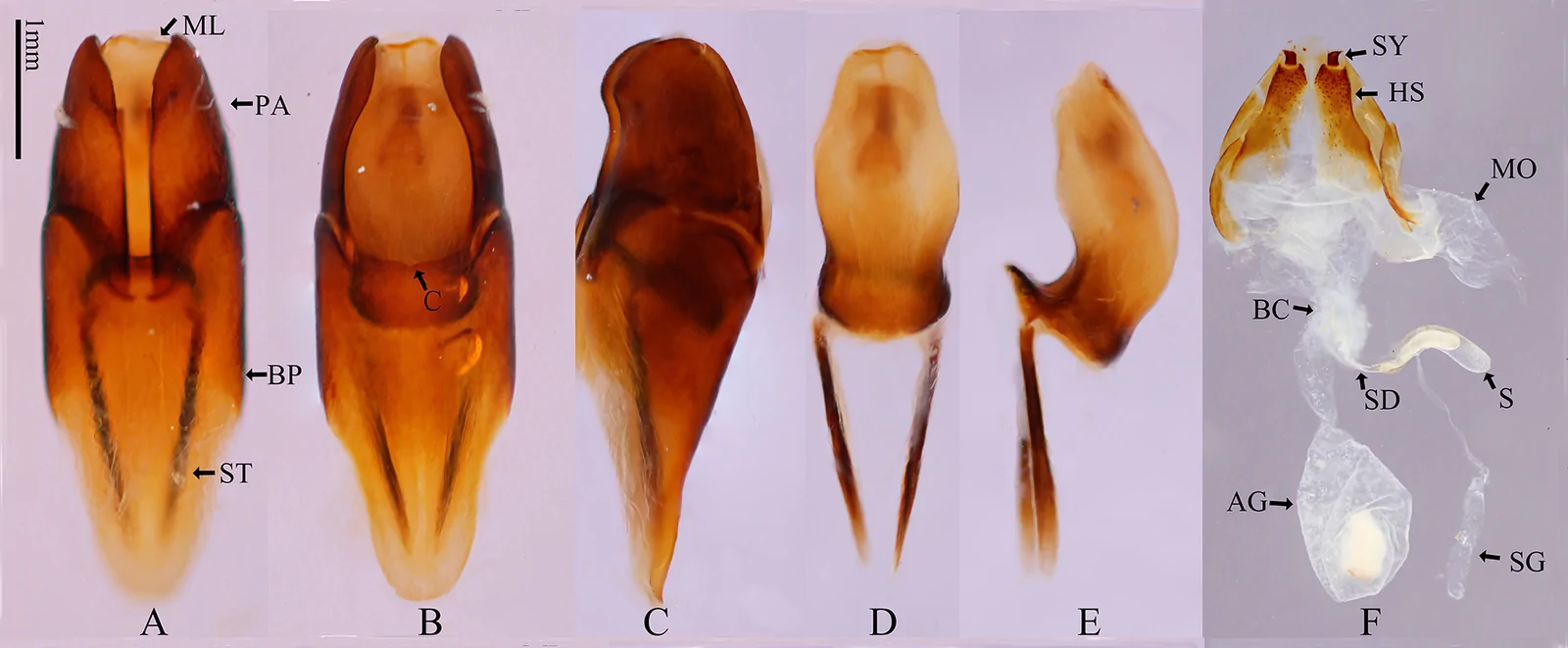
Genitalia of *C. ahualuo* sp. nov. **A–E**. Aedeagus: **A**. Dorsal view; **B**. Ventral view; **C**. Lateral view; **D**. Median lobe, ventral view; **E**. Median lobe, lateral view; **F**. Female genitalia. Abbreviations: ML = median lobe; PA = parameres; C = caudal margin of basal piece of aedeagus; BP = basal piece; ST = strut; SY = styli; HS = hemisternite; MO = median oviduct; BC = bursa copulatrix; SD = spermathecal duct; S = spermatheca; AG = accessory gland; SG = spermathecal gland. Scale bars: 1 mm.

##### Type material.

***Holotype***: China • ♂; Yunnan Province: Weixi County, Mts. near Ahualuo River, elev. 2600 m, in decayed logs, 2024-IV-2, Baijun Li leg., deposited in AHU. ***Paratypes***: China • 4♂, 2♀ Yunnan Province: Weixi County, Zhonglu Township, elev. 2750 m; in decayed logs, 2021-III-5, Yanchen Liu leg., (AHU). China • 3♂ Yunnan Province: Weixi County, Mts. near Ahualuo River, elev. 2800–3000 m, in decayed logs, 2026-V, Yujie Huang leg., (AHU).

##### Description.

**Male** (Figs 1A, 1B, 1D, 1E, 2)**. *Body*** length 13.5–17.0 mm. Body black, labial palps, maxillary palps and legs slightly paler. ***Pronotum*** wider than long, strongly convex, with relatively broad lateral margins. ***Elytra*** with 7 distinct longitudinal striae, humeral angles obtusely rounded. ***Scutellum*** semicircular. ***Legs*** slender; ventral surface of femora with sparse yellow setae; ventral apex of mesotibiae with a yellow setal brush.

***Head*** with vertex bearing a distinct subconical depression anteromedially. ***Labrum*** triangular. ***Mandibles*** slender, longer than head, apex acute, inner margin clothed with yellow setae. Base of ventral margin with a triangular obtuse tooth directed vertically downward, smaller in minor individuals; a very small projection present at approximately apical two-thirds of mandibular length, more distinct in minor individuals. Dorsal margin near base with a large finger-like tooth (Fig. 2), curved upward and directed obliquely forward; one subquadrate obtuse denticle positioned immediately posterior to this large tooth toward base and directed horizontally.

***Aedeagus***. (Fig. 3A–E). Parameres broad and plate-like, with bluntly rounded apices, curved dorsally. Median lobe moderately sclerotized and equal in length to parameres, broadened medially and tapering toward both base and apex. Basal piece stout, longer than parameres. Struts approximately as long as median lobe. Caudal margin of the basal piece of the aedeagus with a shallow median emargination.

**Female**. (Fig. 1C, F). ***Body*** length 10.5–13.5 mm. Dark brown. Mandibles small, shorter than head. Dorsal margin of mandibular base forming a slightly upward-curved arched plate; apex forming an acute triangular denticle, base obtuse and quadrate, middle bearing a very indistinct small denticle, rendering arched plate shallowly trifurcate. Base of ventral margin without a tooth. Anteromedian portion of vertex with a very shallow depression. Pronotum subtrapezoidal, disc distinctly convex and ridge-like, flanked by a small convexity on each side of the elevation. Setae on inner margin of mandibles, mesofemora, and ventral apex of mesotibiae much less conspicuous and dense than in male. Other characters similar to those of male.

***Female genitalia*** (Fig. 3F). Hemisternites subtrapezoidal and plate-like, bearing small styli at apex. Accessory duct long, broad, and apically spherical. Spermatheca slightly sclerotized; spermathecal duct relatively thin and short, distinctly separated from and narrower than spermatheca; spermathecal gland slender.

##### Diagnosis.

**Figure 4.**
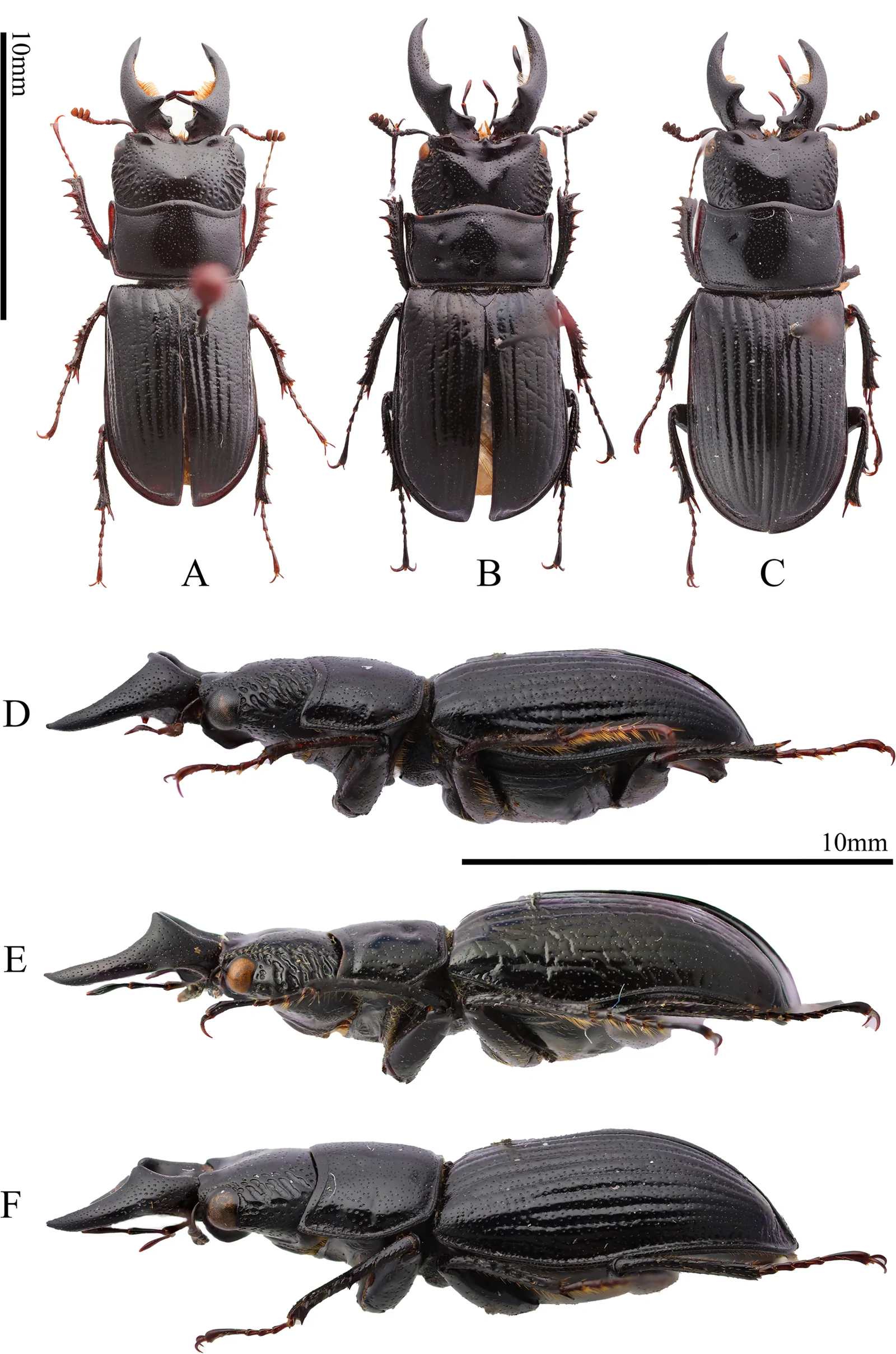
Closely related species in dorsal and lateral views. **A, D**. *C. niger*; **B, E**. *C. sinensis*; **C, F**. *C. yangi*; **A–C**. Dorsal views; **D–F**. Lateral views. Scale bars: 10 mm.

*Ceruchus ahualuo* sp. nov. is similar to *C. niger* (Fig. 4A, D), *C. sinensis* (Fig. 4B, E), and *C. yangi* (Fig. 4C, F) in body size and the density of setae on the mesofemora, but can be distinguished from them by the large finger-like tooth on the dorsal surface near the base of each mandible. This discrete mandibular character was not observed in other examined species of *Ceruchus*. In the male genitalia, the new species differs from *C. yangi* and *C. niger* by the caudal margin of the basal piece of the aedeagus with a shallow median emargination in ventral view (vs. caudal margin of the basal piece forming an M-shaped margin in ventral view; cf. Fig. 3A and Huang et al. 2011: figs 134–139, 164–169). It also differs from *C. sinensis* by the median lobe swollen medially in lateral view (vs. median lobe not swollen medially in lateral view; cf. Fig. 3E and Huang et al. 2011: figs 196–201).

**Figure 5.**
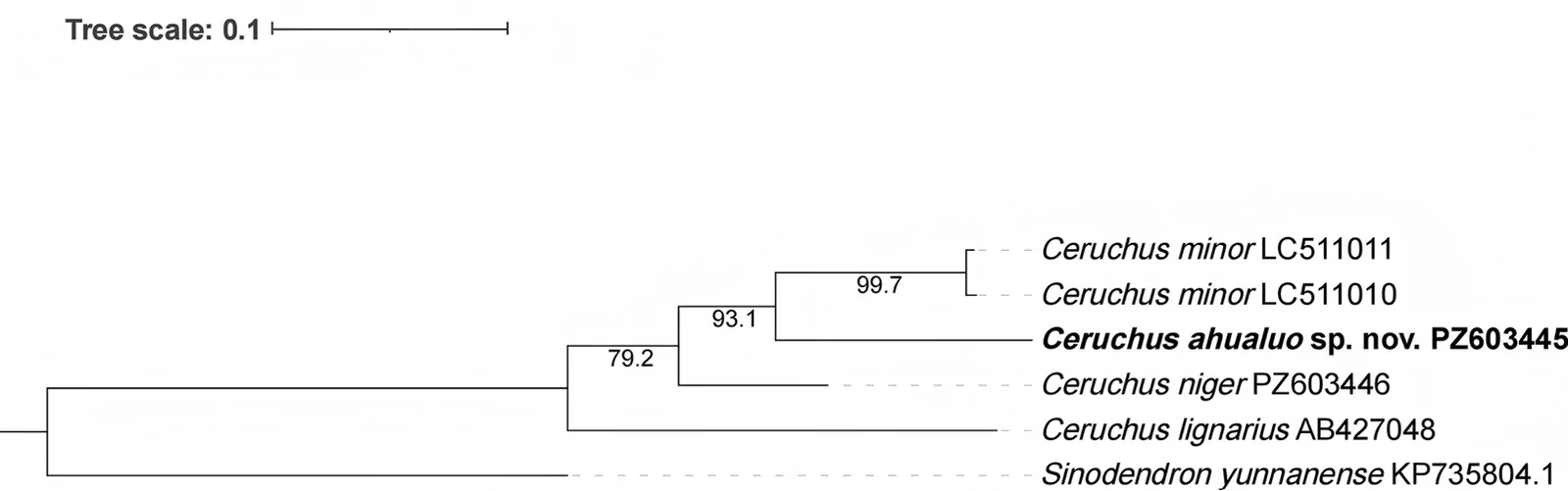
Maximum-likelihood tree based on mitochondrial COI sequences of selected *Ceruchus* species, with *Sinodendron yunnanense* used as the outgroup. Numbers at nodes indicate branch support values. The new species is highlighted in bold.

Given the sympatric occurrence of *C. ahualuo* sp. nov. and *C. niger*, the COI data were used to further assess their separation. In the maximum-likelihood tree based on mitochondrial COI sequences, the new species was recovered as a distinct lineage and was clearly separated from *C. niger* (Fig. 5). The pairwise K2P distance between the two species was 0.1359, falling within the range of interspecific distances observed among the sampled *Ceruchus* species and much higher than the distance between the two *C. minor* sequences (0.0077; Table 2). These molecular results are consistent with the morphological differences described above and provide additional support for separating the new species from the sympatric *C. niger*.

**Table 2.** Pairwise K2P distances among selected *Ceruchus*COI sequences. The new species is indicated in bold.

	**Taxon**	**1**	**2**	**3**	**4**	**5**
1	***Ceruchus ahualuo* PZ603445.1**					
2	*Ceruchus niger* PZ603446.1	**0.1359**				
3	*Ceruchus lignarius* AB427048.1	**0.1823**	0.1741			
4	*Ceruchus minor* LC511010.1	**0.1395**	0.1272	0.1823		
5	*Ceruchus minor* LC511011.1	**0.1395**	0.1256	0.1733	0.0077	

##### Etymology.

The specific epithet ahualuo is a noun in apposition, derived from the name of the type locality of this species.

##### Distribution.

China: Yunnan.

### Key to species of *Ceruchus* MacLeay from China

**Table:** 

1	Lateral margins of pronotum strongly arcuate	***C. luojishanensis***
–	Lateral margins of pronotum subparallel	**2**
2	Mesofemora with dense yellow setae, forming a brush	**3**
–	Mesofemora with sparse yellow setae, not forming a brush	**6**
3	Base of dorsal margin of mandibles with one incurved obtuse tooth	***C. reginae***
–	Base of dorsal margin of mandibles with two incurved obtuse teeth	**4**
4	Mandibles curved	***C. deuvei***
–	Mandibles relatively straight	**5**
5	Obtuse tooth at base of ventral mandibular margin reduced	***C. ganyanae***
–	Obtuse tooth at base of ventral mandibular margin distinct	***C. katerinae***
6	Mandibles robust	**7**
–	Mandibles slender	**8**
7	Anterior margin of mentum straight, not protruding medially	***C. minor***
–	Anterior margin of mentum distinctly protruding medially	***C. niger***
8	Obtuse tooth at base of ventral margin of mandibles reduced	***C. yangi***
–	Obtuse tooth at base of ventral margin of mandibles distinct	**9**
9	Groove formed by punctate line on postocular lateral margin deep, lateral margin of head distinctly undulate	***C. motuoensis***
–	Groove formed by punctate line on postocular lateral margin shallow, lateral margin of head not distinctly undulate	**10**
10	Large tooth on dorsal mandibular margin finger-like, distinctly extending obliquely forward	***C. ahualuo* sp. nov**.
–	Large tooth on dorsal mandibular margin triangular, directed subhorizontally or only slightly obliquely forward	**11**
11	Elytral longitudinal striae indistinct	***C. yingqii***
–	Elytral longitudinal striae distinct	**12**
12	Apices of parameres truncate in lateral view	***C. tabanai***
–	Apices of parameres semicircular in lateral view	***C. sinensis***

## Discussion

Male *Ceruchus* primarily use their broad, thick mandibles for excavating decaying wood rather than for frequent combat. Species of this genus are adapted to cold and temperate climates; these beetles typically inhabit high-altitude areas at low latitudes and low-altitude areas at high latitudes across the Palaearctic and Nearctic regions.

**Figure 6.**
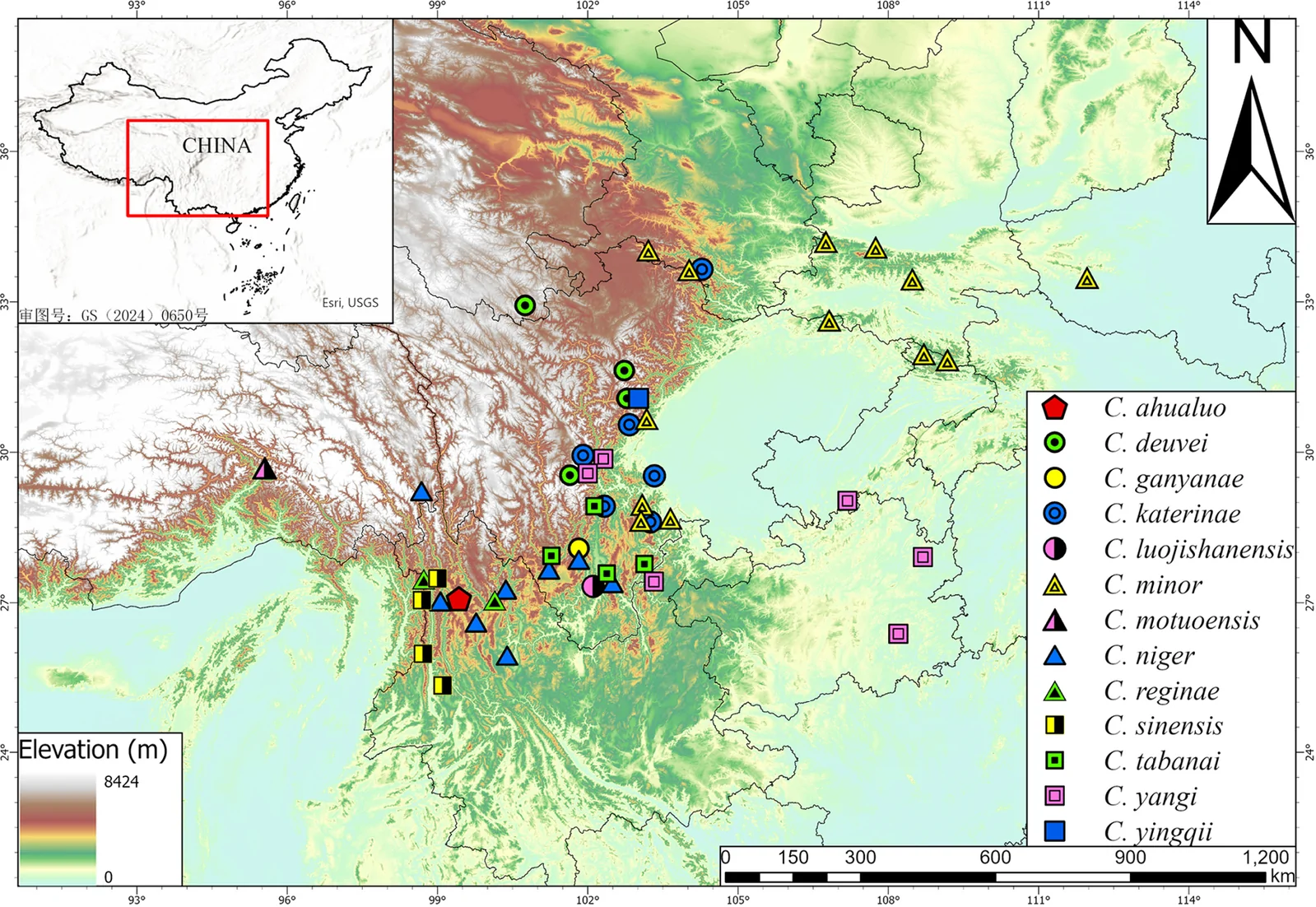
Distribution map of the genus *Ceruchus* in China.

In China, the core distribution of the genus *Ceruchus* is concentrated along the eastern margin of the Qinghai-Tibet Plateau and in the Hengduan Mountains (Fig. 6). Species are densely distributed along these complex north–south-oriented mountain systems. This pattern suggests that the Hengduan Mountains may represent a major centre of diversification or refugium for the genus in East Asia. In addition, certain species (e.g., *C. minor* and *C. yangi*) extend eastward beyond the Hengduan region, dispersing along mountain systems such as the Qinling and Daba Mountains into mid- to high-elevation habitats in central and southern China. Overall, the broad distribution pattern of *Ceruchus* closely follows the major topographic transition zones of China, implying that geographic isolation imposed by complex landscapes (e.g., deep gorges and high mountains), together with associated microhabitat variation, likely played a crucial role in the radiation and present-day distribution of the genus.

Interspecific differences within *Ceruchus* are generally slight, and male polymorphism is not strongly developed. Traditionally, mandibular morphology and the density of setae on the mesofemora have been regarded as characters of high diagnostic value. Based on our dissections, male genitalia may exhibit some degree of variation among different geographic populations, whereas female genitalia appear to show only limited interspecific differentiation. Nevertheless, specific aspects of the aedeagus morphology, such as the parameres, median lobe, and the caudal margin of the basal piece in ventral view, when combined with selected external morphological characters, may still provide useful taxonomic evidence.

Unlike many other lucanid groups, in which male allometry produces extreme intraspecific variation (Rowland and Emlen 2009), *Ceruchus* presents a distinct taxonomic challenge. Specimens of this genus are scarce in the collections available to us, such as *C. ganyanae* and *C. luojishanensis*, and some species are known from only a few records, with very limited material available for examination. As noted by Lim et al. (2012), the prevalence of singletons and rare species poses a fundamental challenge to species delimitation because it restricts the assessment of true intra- and interspecific variation. Consequently, many diagnostic characters still require further evaluation based on additional specimens and, where possible, support from molecular data. Future taxonomic revisions will therefore require broader field surveys to obtain additional specimens, together with rigorous population-level morphometric analyses and molecular phylogenetic evidence, in order to establish a stable taxonomic framework for the genus.

## Supplementary Material

XML Treatment for Ceruchus

XML Treatment for Ceruchus ahualuo

## Additional information

### Artificial Intelligence (AI) use

The authors accept full responsibility for the content of the manuscript, including the disclosure of any use of AI.

Regarding the use of AI in the preparation of this manuscript, the authors declare the following: Gemini.

**Used for**: Language, style and writing.

Gemini was used for English language editing and polishing.

### Author ORCIDs

Y. Huang https://orcid.org/0009-0009-3319-8897

Y. Liu https://orcid.org/0000-0001-6550-1971

Y. Shen https://orcid.org/0009-0009-5153-7151

X. Feng https://orcid.org/0000-0001-6705-9929

X. Wan https://orcid.org/0000-0002-5783-0676

### Data availability

All of the data that support the findings of this study are available in the main text or Supplementary Information.
